# *Impatiens glandulifera* (Himalayan balsam) chloroplast genome sequence as a promising target for populations studies

**DOI:** 10.7717/peerj.8739

**Published:** 2020-03-24

**Authors:** Giovanni Cafa, Riccardo Baroncelli, Carol A. Ellison, Daisuke Kurose

**Affiliations:** 1 CABI Europe, Egham, Surrey, UK; 2 University of Salamanca, Instituto Hispano-Luso de Investigaciones Agrarias (CIALE), Villamayor (Salamanca), Spain

**Keywords:** Balsaminaceae, Chloroplast genome, *Impatiens glandulifera*, Whole genome sequencing, Genome skimming, Phylogenetic analyses

## Abstract

**Background:**

Himalayan balsam *Impatiens glandulifera* Royle (Balsaminaceae) is a highly invasive annual species native of the Himalayas. Biocontrol of the plant using the rust fungus *Puccinia komarovii* var. *glanduliferae* is currently being implemented, but issues have arisen with matching UK weed genotypes with compatible strains of the pathogen. To support successful biocontrol, a better understanding of the host weed population, including potential sources of introductions, of Himalayan balsam is required.

**Methods:**

In this molecular study, two new complete chloroplast (cp) genomes of *I. glandulifera* were obtained with low coverage whole genome sequencing (genome skimming). A 125-year-old herbarium specimen (HB92) collected from the native range was sequenced and assembled and compared with a 2-year-old specimen from UK field plants (HB10).

**Results:**

The complete cp genomes were double-stranded molecules of 152,260 bp (HB92) and 152,203 bp (HB10) in length and showed 97 variable sites: 27 intragenic and 70 intergenic. The two genomes were aligned and mapped with two closely related genomes used as references. Genome skimming generates complete organellar genomes with limited technical and financial efforts and produces large datasets compared to multi-locus sequence typing. This study demonstrates the suitability of genome skimming for generating complete cp genomes of historic herbarium material. It also shows that complete cp genomes are solid genetic markers for population studies that could be linked to plant evolution and aid with targeting native range and natural enemy surveys for biocontrol of invasive species.

## Introduction

*Impatiens glandulifera* Royle (Balsaminaceae), commonly known as Himalayan balsam, is an annual plant species native to the foothill of the Indian and Pakistani Himalayas, where it can be found in mixed plant communities at altitudes from 2,000 to 3,000 m. It was introduced into the UK in 1839 as an ornamental plant (Beerling & Perrins, 1993) and is now considered to be one of the worst invasive, non-native plant species in the UK particularly in riverine habitats (Environment Agency, 2010), although it also flourishes in damp woodlands and waste grounds (Tanner et al., 2014). Himalayan balsam is the tallest annual plant in Europe, attaining a height of 2.5 m, significantly taller than seen in its native range. Each plant can produce up to 2,500 seeds that are forcibly ejected up to 7 m away from the parent plant and able to survive for up to 2 years in the soil seed bank. Seeds which land in rivers are transported downstream to new sites, establishing particularly well in disturbed habitats (Tanner et al., 2014; Tanner, 2017). It forms dense monocultures and outcompetes native plant species (Perrins, Fitter & Williamson, 1993; Hulme & Bremner, 2006; Pattison et al., 2016); this is aided by seed germination early in the growing season, its extremely fast growth rate and potential allelopathic effect on soil mycobiota (Lobstein et al., 2001; Clements et al., 2008; Tanner & Gange, 2013). The showy flowers rich in nectar lure pollinators away from native plants, reducing pollination opportunities (Beans & Roach, 2015). As the plant dies back in the autumn in riverine habitats, banks laid bare have an increased risk of erosion and dead plant material entering the water course can cause blockages leading to flooding. Himalayan balsam stands have also been shown to alter invertebrate communities and negatively affect detritivores, which are reliant on native plant species (Tanner et al., 2013).

Biological control, using a rust fungus (*Puccinia komarovii* var. *glanduliferae*) collected from the native range, is currently being implemented in the UK, but issues have arisen with matching the genotype of the weed with a compatible strain of the rust fungus (Tanner et al., 2015; Varia, Pollard & Ellison, 2016). In order to enhance the likelihood of successful biological control, molecular studies are carried out to better understand the host weed population and its relationship with pathogen genotypes in both the native and introduced ranges of a weed (Gaskin et al., 2011). In the introduced range, it is important to ascertain how genetically diverse the invasive population is. A single genetic entity of a natural enemy may be unilaterally effective if the invasive weed population is genetically uniform (Tomley & Evans, 2004). However, an invasive plant is often introduced on more than one occasion and from different parts of the native range, resulting in increased genetic diversity in the introduced range. This information is particularly important with co-evolved biotrophic pathogens, such as rusts, because they often express intraspecies specificity (Evans, 2002; Ellison, Evans & Ineson, 2004). Hence, more than one strain of a pathogen sourced from the area where the introduction originated may be required.

Previous studies (Kurose, Pollard & Ellison, 2018) sampled leaves from across the native range in India and Pakistan and the introduced range in the UK and Ireland with specimens dating from 1881 to 2016. Six regions of the cp genome were included in this study with a multi-locus sequence typing (MLST) approach, which represented a limited portion of the cp genome of around 2.5% of the complete sequence of ~150 Kb genome. These were *trnL-F*, *atpB*-*rbcL*, *rps16* Intron, *trnG* Intron, *psbA*-*trnH*^(GUG)^ and *rpl32*-*trnL*^(UAG)^ (Kurose, Pollard & Ellison, 2018). The study showed that the investigated specimens segregated into three groups, suggesting that Himalayan balsam in the UK was introduced at least three times from the native range. However, one of the biotypes found in the UK has not been matched in the native range and therefore additional molecular testing is needed for further investigation. Studies targeting complete cp genomes might provide better resolution as they can examine additional genes beyond the six previously investigated by MLST. Surveys targeting the three regions identified (Pakistan, Indian Kashmir and Nepalese border region with India) could provide rust fungus isolates more pathogenic to Himalayan balsam genotypes invasive in the UK. In addition, sequencing of complete cp genomes from historic herbarium material could allow the comparison with recently sampled specimens and gain knowledge to establish the origin of invasive alien weed populations. Herbarium collections have already shed new light on the mechanisms shaping species adaptation and diversification and biodiversity patterns over space and time (Besnard et al., 2018). A specimen stored in an herbarium is an archive document, which proves species distribution in a certain territory and habitat at a certain time (Jukonienė, Subkaitė & Ričkienė, 2019). The use of historic herbarium specimens can inform about long-term effects on plants of at least four of the main drivers of global change: pollution, habitat change, climate change and invasive species (Lang et al., 2019).

Whole organellar genomes such as the cp genome can be obtained by genome skimming. Straub et al. (2012) first showed that “genome skimming” is suitable for the recovery of highly repetitive genome regions such as the ribosomal cluster (rRNA) or organelle genomes and the generation of entire genome data at a relatively low sequence depth. In addition, genome skimming has been used to recover plastid DNA and rRNA from up to 83-year-old herbarium specimens (Zeng et al., 2018). Li et al. (2018) published the first complete cp genomes from the genus *Impatiens* and the monotypic genus *Hydrocera*, the only two genera within the family Balsaminaceae. The study provides the genomic data that clearly positions the Balsaminaceae as sister to the rest of the families of the order Ericales. Chloroplast (cp) genomes of Balsaminaceae are of ~150 Kb and include 110–130 distinct genes, with approximately 80 Protein-coding genes (PCG), 30 tRNA and 4 rRNA genes, consistent with those of other angiosperms (Jansen & Ruhlman, 2012). Most of the genetic regions regularly examined for the phylogeny of *Impatiens* with MLST (Yu et al., 2015; Kurose, Pollard & Ellison, 2018) are in the cp genome, such as the regions *trnL-F*, *atpB*-*rbcL*, *rps16* Intron, *trnG* Intron, *psbA*-*trnH*^(GUG)^ and *rpl32*-*trnL*^(UAG)^.

The objectives of this study were to test low coverage whole genome sequencing (genome skimming) and assembly protocols for the generation of complete cp genomes to identify invasion sources of *I. glandulifera*. Specifically, they were to: (i) determine the possibility of successfully generating a complete cp genome of a 125-year-old *I. glandulifera* herbarium specimen by genome skimming; (ii) determine the complete sequence of the cp genomes of an introduced and a native specimen of *I. glandulifera*; (iii) document pairwise differences between the complete cp genomes of an introduced and a native specimen of *I. glandulifera*.

## Materials and Methods

### DNA isolation and template preparation

DNA was isolated from fresh and herbarium leaves of *I. glandulifera* (Himalayan Balsam). Himalayan Balsam leaves of specimen HB10 were obtained in 2016 from Silwood park, Berkshire, UK (Latitude 51.40756, Longitude −0.64374), dried in silica gel and kept at 4 °C. The other sample HB92, collected from Liddar Valley, Jammu and Kashmir, India (Latitude 33.9131, Longitude 76.4797) in 1893, was obtained from the herbarium of the Natural History Museum, UK (Catalogue Number BM001254006). DNA was isolated from HB10 with DNeasy Plant Mini Kit (Qiagen, Hilden, Germany) and from HB92 with Power Plant Pro DNA Isolation Kit (MO BIO Laboratories Inc., West Carlsbad, CA, USA). DNA extracts were quantified with Qubit 3.0 Fluorometer (Thermofisher Scientific, Loughborough, UK).

### Illumina library preparation

Genomic DNA was fragmented after quantification and quality assessment for library preparation. A total of 100 ng of DNA were diluted in 50 µL of 10 mM TRIS–HCl pH 8.5—Ethanol 20% (v/v) solution (Sigma–Aldrich Merck, Gillingham, UK) and sheared with Covaris M220 Focused-ultrasonicator (Covaris Ltd., Brighton, UK) with the following settings for a 350 bp insert size: duty factor (%) 20.0, peak power 50.0, cycles/Burst 200 and duration of 65 s at 20 °C. Libraries were prepared with a Truseq Nano DNA Library prep kit (Illumina, Cambridge, UK), according to the manufacturers’ instruction. Steps included fragmentation, repair ends and library size selection, addition of adenylate 3′ ends, ligation of adapters, enrichment of DNA Fragments, normalization and pool. The paired end libraries were generated with the Illlumina MiSeq at CABI (Egham, UK), with an average insert size of 350 bp and run on an Illumina MiSeq 250-PE V2 Cartridge (500 cycles) (Illumina, Cambridge, UK).

### Cp genome sequence assembly and annotation

High quality reads were filtered from Illumina raw reads with the MiSeq Reporter analysis software v3.0 (Illumina, Cambridge, UK). Assembly was performed with SPAdes v3.11.1 (Bankevich et al., 2012). Scaffolds belonging to the cp genome were identified by BLAST searches to a custom database built with the cp genome of *Impatiens pinfanensis* (MG162586). Also, an assisted assembly was performed on *Impatiens pinfanensis* (MG162586); raw reads were mapped to the reference genome using Bowtie2 v2.3.5.1 (Langmead & Salzberg, 2012), the process was then repeated on the obtained scaffolds in order to fill the gaps. The final fragment was circularized using Geneious v.2019.0.3 (Biomatters Ltd., Auckland, New Zealand). The annotations of the complete cp genomes were performed with GeSeq (Tillich et al., 2017). The circular cp genome maps were generated with the online tool OGDraw v1.2 (Lohse, Drechsel & Bock, 2007) with default settings. The complete cp genome sequences were submitted to GenBank database, accession numbers *I. glandulifera* HB10 (MK358446) and *I. glandulifera* HB92 (MK358447). The two reference genomes of *I. pinfanensis* (MG162586) and *Hydrocera triflora* (MG162585) (Li et al., 2018) represent the two genera of the Balsaminaceae family.

Whole genome alignment was performed on annotated merged contigs with progressive MAUVE 2.4.0 (Darling, Mau & Perna, 2010) and rearrangements were visualized using Geneious v.2019.0.3 (Biomatters Ltd., Auckland, New Zealand).

### Phylogenetics

Nucleotide sequences produced in this work as well as references previously published (Fujihashi, Akiyama & Ohba, 2002; Lim et al., 2014; Aust, Ahrendsen & Kellar, 2015; Li et al., 2018) were aligned using MAFFT v.7.407 (Katoh & Standley, 2013), manually checked and if necessary, trimmed on both ends to have comparable nucleotides. Sequence alignments (1,262 ± 0 bp) were exported to MEGA7 (Kumar, Stecher & Tamura, 2016) and the best-fit substitution model using Maximum Likelihood statistical method was calculated (T92 + G + I) and used in MrBayes v.3.2.6 (Ronquist & Huelsenbeck, 2003). The Markov chain Monte Carlo (MCMC) algorithm was performed to generate phylogenetic trees with Bayesian posterior probabilities. Four MCMC chains were run simultaneously for random trees for 5,000,000 generations. Samples were taken every 1,000 generations. The first 25% of trees were discarded as burn-in phase of each analysis and posterior probabilities were determined from the remaining trees. A second phylogenetic tree was built using Fast Tree v.2.1.5 (Price, Dehal & Arkin, 2010) as implemented in Geneious v.2019.0.3 (Biomatters Ltd., Auckland, New Zealand). The trees obtained showed the same topology and therefore only the Bayesian inference phylogenetic tree is shown in results.

## Results

DNA isolation and sequencing of the complete chloroplast (cp) genome of the 125-year-old herbarium specimen of *I. glandulifera*.

DNA yield was of 33.0 ng/µL for *I. glandulifera* HB92 (native herbarium specimen) as compared to the fresh leaves of *I. glandulifera* HB10 (introduced specimen) with 99.2 ng/µL. The number of raw DNA reads after sequencing with an Illumina MiSeq was of 1,104,294 for HB92 and of 1,696,776 for HB10, with the quality control showing no differences between the libraries of HB92 and HB10. On average, lower coverage was obtained for HB92 than HB10, with an average coverage of the nodes including fragments of the cp genome of 15X and of 45X, respectively.

### The *I. glandulifera* complete cp genome structure and gene content

**Figure 1:**
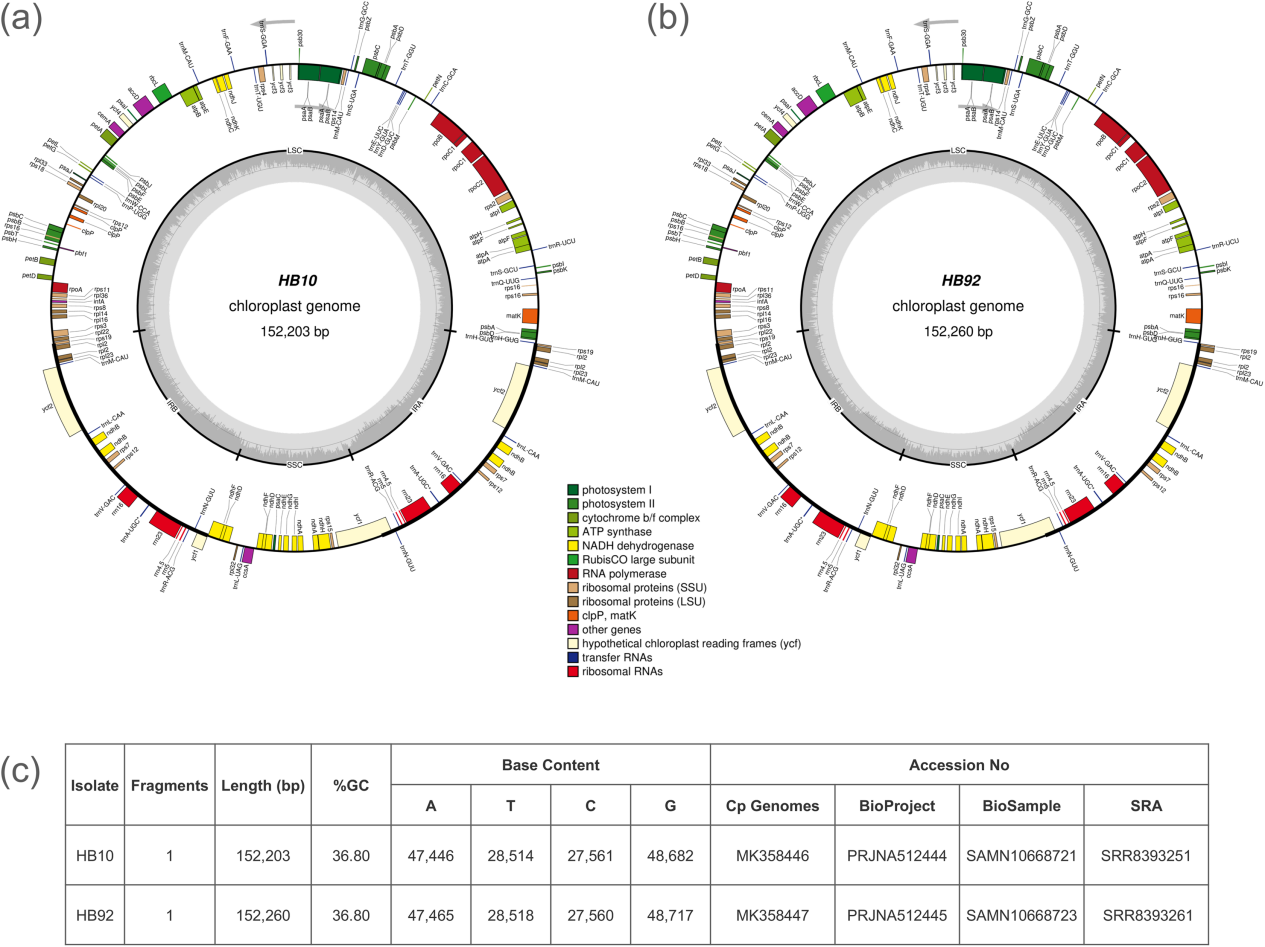
Gene maps of *Impatiens glandulifera* chloroplast (cp) genome. Genes drawn inside of the circle are transcribed counterclockwise, while genes outside the circle are transcribed clockwise. Gene functional groups are color-coded. The dark gray area in the inner circle corresponds to GC content while the light gray corresponds to the adenine-thymine (AT) content of the genome: (A) isolate HB10; (B) isolate HB92; (C) summary of sequencing and GenBank accession data.

Unbalanced distribution of coverage was generated by the assembler software in the scaffolds of the cp genome. A reference assisted approach was necessary to obtain the complete cp genome. The cp genomes of the two specimens were fragmented in three scaffolds, with one of the three having double value of average coverage. For example, HB10 had the three scaffolds of 83.2 Kb, 18.7 Kb and 17.7 Kb with an average coverage of 44X, 94X and 46X, respectively. The final cp genomes could be represented as circular molecules and were of 152,203 bp for the specimen HB10 of *I. glandulifera* (the introduced specimen) and of 152,260 bp for the specimen HB92 (the native herbarium specimen) (Fig. 1). The genomes contained 86 protein-coding genes (PCG), 31 transfer RNA and four ribosomal RNA genes.

### The *I. glandulifera* cp genomes of the native and introduced specimens

**Figure 2:**
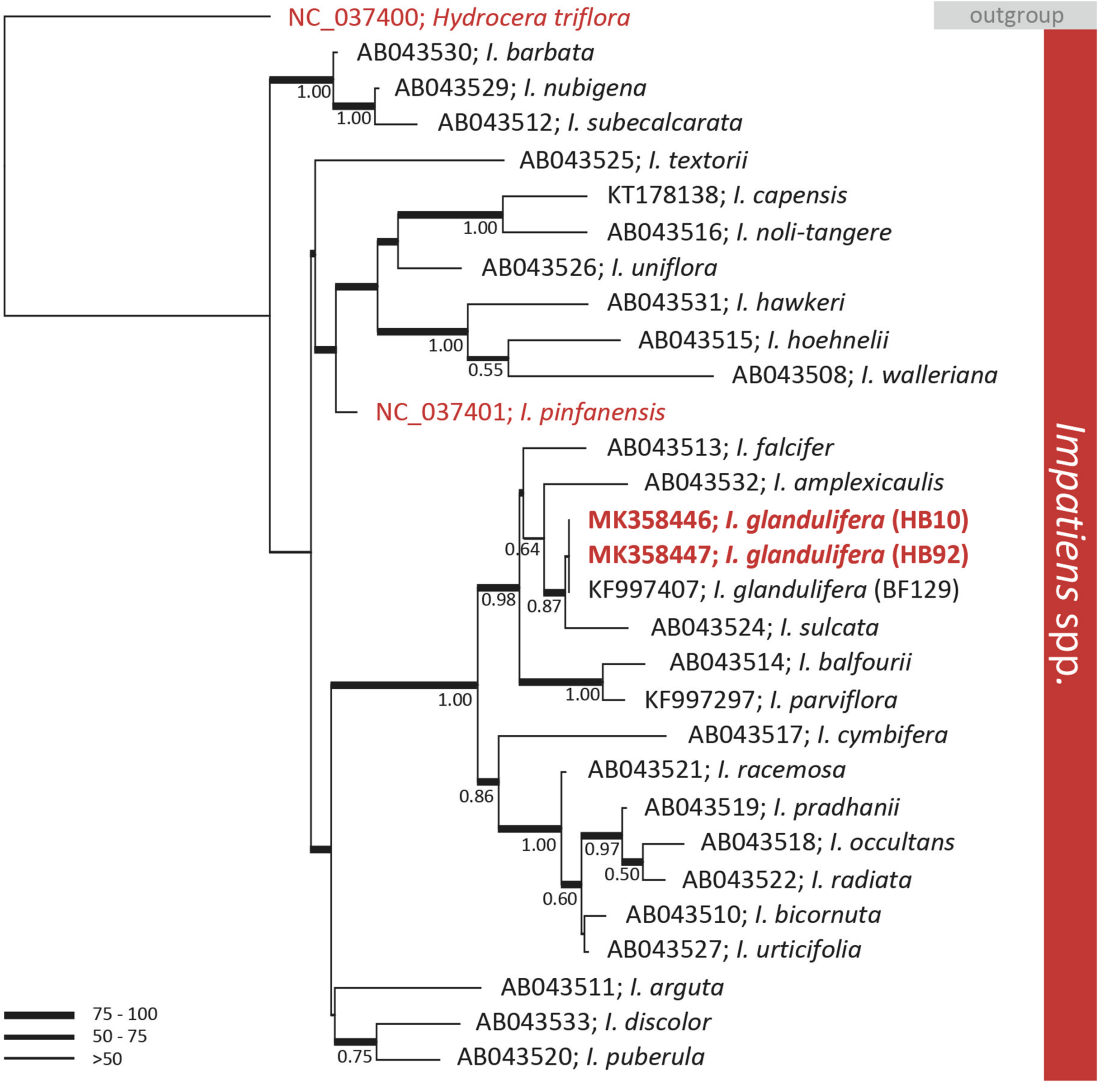
Phylogenetic tree of the genus *Impatiens*. Bayesian inference phylogenetic tree reconstructed from the alignment of 29 ribulose-1, 5-bisphosphate carboxylase large subunit (*rbcL*) sequences belonging to the *Impatiens* genus (Fujihashi, Akiyama & Ohba, 2002; Lim et al., 2014; Aust, Ahrendsen & Kellar, 2015; Li et al., 2018). The tree was rooted with the closely related species *Hydrocera triflora*. ML bootstrap values are represented with branch thickness while Bayesian posterior probabilities (BPP) values (above 0.50) are reported next to the node. Reference sequences from GenBank and taxonomic designation are reported. In red are highlighted sequences retrieved by full chloroplast genomes while in bold those produced in this work.

A preliminary phylogenetic investigation was carried out on the assembled genomes by extracting the *rbcL* genes identified by BLASTn and comparing those with publicly available data. The *rbcL* gene is a key marker universally employed in MLST. BLAST analyses of the gene allowed the identification of two publicly available complete cp genomes closely related to *I. glandulifera*. These two genomes were of *I. pinfanensis* (MG162586) and of *H. triflora* (MG162585) (Li et al., 2018). The analysis of the key MLST gene *rbcL* did not discriminate among geographic isolates of *I. glandulifera*. The *rbcL* sequence of the native and introduced to the UK specimens were identical (Fig. 2). However, the gene *rbcL* did differentiate *I. glandulifera* and *I. pinfanensis*, where 1.1% of the bases were different (14/1,262 bp). The overall diversity of *rbcL* among *I. glandulifera*, *I. pinfanensis* and *H. triflora* was of 2.9% of the bases (37/1,262 bp).

Similarly to the analysis of *rbcL*, complete cp genomes were most similar between the two *I. glandulifera* genotypes and least similar between genera. However, unlike *rbcL*, the cp genomes of HB10 and HB92 were not identical. A small percentage of the bases (206/152,301 bp-0.14%) were variable between the isolates HB10 and HB92, while 4.85% of the bases (7,480/154,134 bp) were variable between the two *I. glandulifera* (HB10 and HB92) as compared to *I. pinfanensis*. Finally, the diversity among the two of *I. glanfulifera* HB10 and HB92, *I. pinfanensis* and *H. triflora* was of 9.54% of the bases (14,958/156,779 bp). Pairwise similarity (Table 1) of the complete cp genomes HB10 and HB92 showed 206 differences, which included multiple substitutions, insertions and deletions for a total of 97 variable sites 27 sites differed within 14 genes (Table S1), while 70 sites differed in intergenic regions (Table S2). Intragenic variability of key genes of MLST genes was further investigated. For example, no differences were found for *rbcL*, while the gene *matK* was different for the two specimens with a substitution of C in HB10 to T in HB92 in position 2,600 of the aligned genomes (Table S1).

**Table 1:** Pairwise similarity of *Impatiens glandulifera* cp genomes HB10 (Silwood park, Berkshire, UK), HB92 (LiddarValley, Jammu and Kashmir, India), and references *I. pinfanensis* and *Hydrocera triflora*.

Pairwise similarity (%)	*I. glandulifera* HB10	*I. glandulifera* HB92	*I. pinfanensis*	*H. triflora*
*I. glandulifera* HB10	100.00	99.87	95.21	92.64
*I. glandulifera* HB92		100.00	95.24	92.68
*I. pinfanensis*			100.00	92.58
*H. triflora*				100.00

Additional synteny analysis with the Mauve multiple-genome alignment was undertaken and showed that the four genomes had the same structure and organization (Fig. S1).

## Discussion

This study demonstrates the success of low coverage whole genome sequencing (genome skimming) for generating complete chloroplast (cp) genomes of historic herbarium material and the suitability of the method to document differences between the complete cp genomes of the 125-year-old herbarium specimen with an introduced specimen of *I. glandulifera*. Historic herbarium specimens contain a large repository of historical and geographical information (Crawford & Hoagland, 2009) and contribute to understanding invasion dynamics and developing management strategies (Lang et al., 2019). The fight against invasive species can benefit from molecular studies targeting host weed populations (Gaskin et al., 2011) and support successful biocontrol with a better understanding of the sources of introductions. The typical maternal inheritance of cpDNA in angiosperms help to retain any genetic structure that may have originated with introductions, when compared to nuclear markers that are subject to gene flow via both pollen and seeds (Gaskin, Zhang & Bon, 2005). In this study, the complete cp genome of a 125-year-old herbarium specimen from the native range of *I. glandulifera* was successfully sequenced and used to document pairwise differences with the complete cp genome of an introduced specimen of *I. glandulifera*. The two complete cp genomes were double-stranded molecules of 152,260 bp (HB92, native herbarium specimen) and 152,203 bp (HB10, introduced specimen) in length and when aligned and mapped with two closely related genomes used as references, showed a total variability of 97 sites.

A comparison of genome skimming against MLST was carried out. MLST entails large amount of PCR and Sanger sequencing efforts to characterize limited portions of cp genomes. Specifically, Kurose, Pollard & Ellison (2018) investigated a portion of the cp genome of around 2.5% of the complete sequence of ~150 Kb and showed that 1.29% of the bases (50/3,865 bp) were variable among 52 samples of Himalayan balsam collected from both the native and introduced range. A total of 32 variable sites were found among them. In the same specimens investigated in this study, HB10 and HB92, six characters differed over a total of 3,852 bp, specifically, one in *rps16* Intron, two in *trnG* Intron and three in *rpl32*-*trnL*^(UAG)^ (Kurose, Pollard & Ellison, 2018). Genome skimming generates complete cp genomes and reveals significantly more variability than MLST with limited technical and financial investment. We estimate that, in this study, the number of informative sites per unit of information was more than 16 times higher for genome skimming than MLST and that the cost per unit of information was more than 100 times cheaper for genome skimming than MLST. The former generates entire genomes, while the latter captures genes or fractions of genes.

Better information on the variability in invasive plant genotypes could improve the use of natural enemies such as the rust fungus *Puccinia komarovii* var. *glanduliferae*, which has great potential as a biocontrol agent of Himalayan balsam (Gaskin et al., 2011; Tanner et al., 2015). Molecular studies targeting the host weed population in the UK could benefit from additional investigation of variability, as variation in the susceptibility of UK populations to the strains of the rust that have been released is reducing the potential efficacy of the biological control strategy. Fast and cheap approaches such as MALDI-TOF MS can be used for the identification of *I. glandulifera* and closely related *Impatiens* spp. to match the optimal rust based biological control agent (Reeve, Pollard & Kurose, 2018). However, the latter approach is described for glasshouse-grown fresh plants and may not be suited for dry specimens.

In this work, we successfully sequenced, assembled and annotated, the complete cp genomes of a 125-year-old herbarium specimen as well as a relatively recently dried leaf specimen from a UK population. The method showed the ability to successfully sequence complete cp genomes from an historic herbarium specimen and facilitated the direct comparison with a recently sampled specimen. Comparison of complete cp genomes reveal more variable sites than a classical MLST approach and provide a platform for further molecular studies targeting the host weed populations of *I. glandulifera*. Phylogenetics can complement the investigation by enabling comparison of multiple cp genomes and therefore we encourage the use of this approach so that large numbers of complete cp genomes can become publicly available and contribute to increased knowledge of the genetic variation of invasive species at the population level.

A better resolution of the origins of invasive plant populations has implications for the selection of co-evolved and virulent pathogens (Goolsby et al., 2006; Prentis et al., 2009). As cp genomes are effective genetic markers (Gaskin, Zhang & Bon, 2005), the investigation of their variability impacts biocontrol strategies that can be better targeted and tailored to host weed populations (Tanner et al., 2015; Varia, Pollard & Ellison, 2016). This approach provides data that support complete organellar genomes as a cheap and effective genetic markers for larger case studies that aim to study population genetics. The successful sequencing of complete cp genomes from historic herbarium specimens, which proved to be comparable to recently sampled specimens, might be valuable for establishing the origin of invasive alien weed populations and aiding natural enemy surveys in the native range.

## Conclusions

The complete cp genome sequences of two isolates of *I. glandulifera*, the first two sequenced members of the species, were assembled, annotated and analyzed in this study. The genome structure, gene content and gene order were similar in the two isolates. Complete cp genomes were used to investigate the variability of a 125-year-old herbarium specimen collected from the native range and a 2-year-old specimen from invasive UK field plants. Low coverage whole genome sequencing (genome skimming) was suitable for sequencing complete cp genomes of herbarium material and proved to be more informative than classical MLST. The complete cp genomes generated in this study are of critical importance to increase the number of sequence data available for the Balsaminaceae family and provide a basis for further research to tackle the rapid spread of *I. glandulifera*.

## Supplemental Information

10.7717/peerj.8739/supp-1 Supplemental Information 1 Intragenic variability of the two specimens of *Impatiens glandulifera* HB10 (Introduced UK specimen) and HB92 (native India specimen).

10.7717/peerj.8739/supp-2 Supplemental Information 2 Intergenic variability of the two specimens of *Impatiens glandulifera* HB10 (Introduced UK specimen) and HB92 (native India specimen).

10.7717/peerj.8739/supp-3 Supplemental Information 3 Synteny and rearrangements in four Balsaminaceae chloroplast genomes with Mauve multiple-genome alignment. Aligned genomes reflect the level of sequence similarity, and lines linking blocks represent homology. Nucleotide positions are indicated by numbers above each genome, and white regions indicate element specific to a genome.

10.7717/peerj.8739/supp-4 Supplemental Information 4 Sequencing data.

## Additional Information and Declarations

### DNA Deposition

The following information was supplied regarding the deposition of DNA sequences: The sequences are available at GenBank: MK358446 and MK358447.

### Data Availability

The following information was supplied regarding data availability: The data is available at SRA: SRR8393251 and SRR8393261.
