# Supplementary figures and images for: Impatiens glandulifera (Himalayan balsam) chloroplast genome sequence as a promising target for populations studies
Source: PeerJ. 2020 Mar 24;8:e8739. doi: 10.7717/peerj.8739 (PMC7100601; doi:10.7717/peerj.8739)

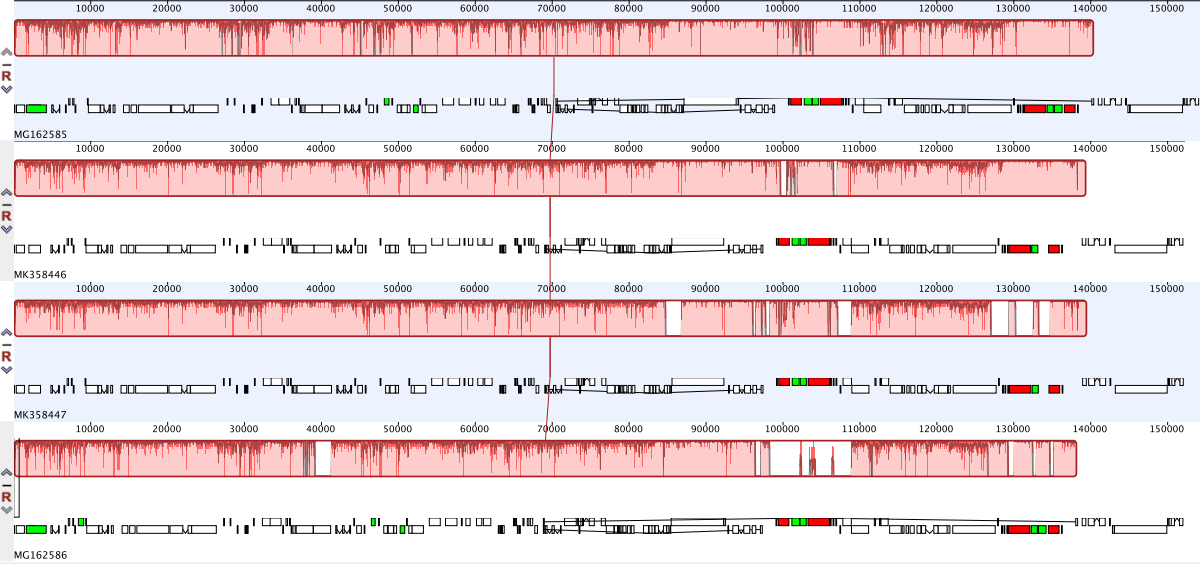

Supplement: Supplemental Information 3 — Aligned genomes reflect the level of sequence similarity, and lines linking blocks represent homology. Nucleotide positions are indicated by numbers above each genome, and white regions indicate element specific to a genome. [file peerj-08-8739-s003.pdf]
